# In Memoriam—Drs. S. A. Seward and A. J. Brewster

**Published:** 1897-06

**Authors:** 


### IN MEMORIAM—DRS. S. A. SEWARD AND A. J. BREWSTER.

The Central New York Homeopathic Medical Society mourns the loss of its highly honored members, Drs. Stephen A. Seward, who died at his home in Syracuse, N. Y., on February 8th, 1897, at the age of eighty-seven years, and Dr. A. Judson Brewster, who died also at his home in the above-named city, on February 20th, 1897, at the age of seventy-two years.

As "the fatal asterisk" is set against the names of the newly-deceased and lamented physicians, the Society recalls, with sorrow, its long death roll, and the personalities of its other members, who, since its organization in the early fifties, have passed over to "the majority:" Drs. Clary, Bigelow, Hoyt, Mera, Spooner, Miller, Brown, Southwick, Benson, Potter, Munger, Hawley, McManus, Richards, Pool, Gardner, and Wells.

Therefore, at a moment so fraught with memories at once painful and consoling the Society has adopted the following resolutions:

*Resolved*, That the Central New York Homeopathic Medical Society has lost, in the death of Dr. Stephen A. Seward and Dr. A. Judson Brewster, the services of skillful physicians, the wise counsels of sincere friends, and the example of men of honor whom it will ever hold in grateful and loving memory.

*Resolved*, That the deep sympathy of this Society be extended to the family and the relatives of its deceased members, Dr. Seward and Dr. Brewster.

*Resolved,* That these resolutions be placed on the records of the Society, and be published in the *Hahnemannian Advocate* and THE HOMEOPATHIC PHYSICIAN, and that copies of them be sent to the families of Drs. Seward and Brewster.

T. DWIGHT STOW,
E. B. NASH,
S. L. GUILD-LEGGETT,
*Committee.*

